# Data mining of audiology patient records: factors influencing the choice of hearing aid type

**DOI:** 10.1186/1472-6947-12-S1-S6

**Published:** 2012-04-30

**Authors:** Muhammad N Anwar, Michael P Oakes

**Affiliations:** 1 Department of Computing, Engineering & Technology, University of Sunderland, St Peter's Way, Sunderland, SR6 0DD, UK

## Abstract

**Background:**

This paper describes the analysis of a database of over 180,000 patient records, collected from over 23,000 patients, by the hearing aid clinic at James Cook University Hospital in Middlesbrough, UK. These records consist of audiograms (graphs of the faintest sounds audible to the patient at six different pitches), categorical data (such as age, gender, diagnosis and hearing aid type) and brief free text notes made by the technicians. This data is mined to determine which factors contribute to the decision to fit a BTE (worn behind the ear) hearing aid as opposed to an ITE (worn in the ear) hearing aid.

**Methods:**

From PCA (principal component analysis) four main audiogram types are determined, and are related to the type of hearing aid chosen. The effects of age, gender, diagnosis, masker, mould and individual audiogram frequencies are combined into a single model by means of logistic regression. Some significant keywords are also discovered in the free text fields by using the chi-squared (χ^2^) test, which can also be used in the model. The final model can act a decision support tool to help decide whether an individual patient should be offered a BTE or an ITE hearing aid.

**Results:**

The final model was tested using 5-fold cross validation, and was able to replicate the decisions of audiologists whether to fit an ITE or a BTE hearing aid with precision in the range 0.79 to 0.87.

**Conclusions:**

A decision support system was produced to predict the type of hearing aid which should be prescribed, with an explanation facility explaining how that decision was arrived at. This system should prove useful in providing a "second opinion" for audiologists.

## Background

This research looks for factors influencing the choice between two common hearing aid types: BTE (worn behind the ear) or ITE (worn in the ear). This choice is typically made by audiology technicians working in out-patient clinics, on the basis of audiogram results and consultation with the patient. In many cases, the choice is clear cut, but at other times the technicians might benefit from a second opinion given by an automatic system with an explanation of how that second opinion was arrived at. The production of such a decision support system is the main goal of this paper. Our data set is unusual in that ITE hearing aids are not generally available on the British National Health Service in England, as they are more expensive than BTE hearing aids. However, both types of aid are prescribed at James Cook University Hospital in Middlesbrough, UK. The data, collected between 1992 and 2001, consists of the following types of records:

• Audiograms (graphs of the auditory thresholds, or faintest sounds audible to the patient at six different pitches or frequencies, where 0 shows perfect hearing and higher thresholds show impaired hearing), e.g., 40, 35, 35, 35, 85, 70, 15, 20, 20, 30, 55, where the first six values are AC (air conduction) and the last five are for BC (bone conduction). AC is measured by placing headphones over the ears, and determines the overall level of hearing. BC is measured by placing the sound source tightly on the mastoid bone behind the ear, and measures the level of hearing of the inner part of the ear. A constraint on the data is that BC must always be the same or better than AC. The difference between the AC and the BC is called the air-bone gap, and measures the hearing ability of the middle and outer parts of the ear.

• Categorical data (such as gender, diagnosis and hearing aid type), e.g., M, TINNITUS, BE18.

• Brief free text notes made by the technicians, e.g., IMPS. TAKEN FOR BINAURAL AIDS., where IMPS is an abbreviation for "impressions", and BINAURAL means "worn in both ears".

## Methods

### Principal component analysis on audiograms

This section describes how a Principal Component Analysis (PCA) was performed on the set of 11,462 audiograms where all AC and BC thresholds for the right ear were recorded, to determine the main audiogram types found among hearing aid users. The rows of our input matrix were the individual audiograms for the right ear, while the 11 columns were for six air conduction and five bone conduction thresholds. Although the patients were originally tested at 11 frequencies, the principle of PCA is that certain frequencies tend to vary together, and thus can be grouped into a smaller number of underlying variables called principal components (PC). Each PC has a set of coefficients in the range -1 to +1, corresponding to the degree of influence of each of the original thresholds on that PC, given in Table 1.

**Table 1:** Component coefficient vectors of PCA

	PC1	PC2	PC3	PC4
AC250	-0.3001	-0.3811	0.2988	-0.1677
AC500	-0.3218	-0.3619	0.2754	-0.0166
AC1000	-0.3410	-0.1999	0.2427	0.2643
AC2000	-0.3436	0.1440	0.1910	0.2697
AC4000	-0.3031	0.3673	0.2409	-0.1742
AC8000	-0.2722	0.3186	0.2629	-0.4684
BC250	-0.2510	-0.2304	-0.4890	-0.5087
BC500	-0.2942	-0.2404	-0.4152	-0.0846
BC1000	-0.3189	-0.0760	-0.3052	0.3595
BC2000	-0.3028	0.2699	-0.2419	0.4088
BC4000	-0.2516	0.4870	-0.2219	-0.1299

A method was devised for converting PCA scores to typical audiogram types, which is an approximation only. It assumes that the PCA scores are directly related to audiogram thresholds, whereas in reality they measure different things: PCA measures the importance of a threshold in distinguishing audiograms, while audiograms measure the degree of hearing loss at the same frequency. The range of PCA scores is -1 to +1, while the range of audiogram thresholds is 0 to 120 dB. If we assume that the relationship is linear, then for each frequency a PCA value of -1 corresponds to an audiogram threshold of 0, and a PCA value of 0 = an audiogram threshold of 60, and a PCA value 1 = an audiogram threshold of 120. The formula relating the two was:

(1) Audiogram_threshold=60+(60×PCA_score).

Each individual patient audiogram was classified into one of the main audiogram types identified, according to least Euclidean distance. A chi-squared test was then performed to determine whether there was any association between the audiogram class of each patient and the type of hearing aid worn. This test was done on the set of 7,437 records where all AC and BC thresholds were available for the right ear, and either a BTE or an ITE aid was specified. In the final logistic regression model, rather than simply using the identified broad audiogram types, each individual hearing threshold was used.

### Use of the chi-squared test to discover other factors related to hearing aid type

In the previous section, it was shown that the choice of hearing aid type was related to the shape of the audiogram. This section describes how the simple chi-squared test was used to discover which of the category data fields were significantly associated with the choice of hearing aid type, and also to discover free-text keywords which were significantly associated with either BTE or ITE hearing aids.

To discover those free-text keywords which were significantly associated with either BTE or ITE hearing aids, first a large contingency table was created where the rows stood for hearing aid type, while each column stood for a candidate keyword (one of 664 distinct words found to have occurred at least once in the free-text fields of the entire record set). The observed value in each cell was the number of times that word had been found in the free-text fields of patients with that type of hearing aid. This table (Table 2) had an overall chi-squared value of 5421.84 for 663 degrees of freedom, giving *p < 0.001*. This data showed, with 99.9% confidence that these free text words were not randomly distributed, but some text words are associated with hearing aid type. To find the association between individual free-text words and hearing aid type, the quantity *(O - E)^2 ^/ E *was examined to rank the keywords according to importance. Some of the keywords in Table 3, were stemmed forms such as 'reshel' for 'reshell' and 'tinnitu' for 'tinnitus', since all the text was passed through Porter's stemmer [1] for the removal of grammatical endings. Some significant keywords were abbreviations, such as IMP for 'impression'. Dictionaries were not used to group terms with the same meaning (synonyms), instead using only the surface form of words because the procedure of keyword selection to was made as automatic as possible. It was assumed that the text notes used in the database were homogeneous, where the various technicians were consistent in their use of terminology and abbreviations. Although it was not done, consistency in writing conventions could have been verified manually, since the identity of the technician is given in one of the record fields. Similarly, it was assumed that two technicians treating the same patient would produce identical audiograms and choose the same hearing aid type. To do otherwise would have resulted in excessive subdivision of the data set.

**Table 2:** Observed and expected frequencies for ITE/BTE aid with gender

Hearing aid type	Male	Female	Row total
BTE	3196 (3369.38) [8.92]	3850 (3676.62) [8.17]	7046
ITE	3647 (3473.62) [8.66]	3617 (3790.38) [7.93]	7264
Column total	6843	7467	14310

**Table 3:** Most significant positive and negative keywords in records with BTE/ITE aid [11]

	Positive keywords	Negative keywords
BTE	mould, be34, map, gp, 92, audio, inf, be52, ref, staff, reqd, be36, contact	fta, reshel, appt, it, nn, nfa, 2001, rev, lacquer, hn, km, imp, review, 2000
ITE	fta, reshel, appt, it, nn, nfa, 2001, rev, lacquer, hn, km, imp, review, 2000, nh, vent, progress, aid, dt, taken	mould, be34, map, gp, 92, audio, inf, be52, ref, staff, reqd, be36, contact, tri, n, order

### Logistic regression (LR) model for ITE/BTE right ear hearing aids

Having determined that audiogram frequencies, gender, presence of tinnitus, use of tinnitus masker, age, mould and certain keywords were all associated with the decision between fitting a BTE or an ITE aid, all these factors were combined into a single logistic regression model. The input to the model was the matrix of patient data, where columns corresponded to attributes and rows corresponded to individual patient records. The output was a formula [2] in the form:

(2) $$L=\text{log}\left(p/\left(1-p\right)\right)=b_{0}+b_{1}x_{1}+b_{2}x_{2}+...+b_{k}x_{k}.$$

In our case p is the probability that the patient should be fitted with an ITE aid, while (1- p) is the probability that the patient should be given a BTE aid. b_0 _is a constant, and b_1 _to b_k _are called the coefficients of the model. The values x_1 _to x_k _are all either 1 or 0, depending on whether a given attribute in the patient's record is present or absent. The overall value L is greater than 0 if it is more likely that the patient should be given a BTE aid, while it is less than 0 if it is more likely that the patient should be given an ITE aid.

Before performing the logistic regression, the actual set of records was divided into two parts, one containing 80 percent (5,736) of the records and the other containing the remaining 20 percent (1,433) of these records. The 80 percent subset was used as the training set for model construction, and the remaining records for testing the model. The sampling method was to extract every fifth record for testing. The logistic regression was performed on the records which had all fields filled for the right ear: AC (air conduction) and BC (bone conduction) thresholds, gender, age and text keywords (5,736 records), of which 128 also had non-null entries for diagnosis, 98 had non-null entries for masker, and 3983 had non-null entries for mould. This data was converted into discrete numeric values as inputs to the model, as follows: For AC thresholds below the first quartile (40 db) a value of 0; for thresholds between the first and second quartile (55 dB) a value of 1; for thresholds between the second and third quartile (75 dB) a value of 2; and for thresholds above the third quartile a value of 3. The same method was used to assign values for BC thresholds and age, except in that the quartile thresholds were 25, 40 and 55 dB and 60, 70 and 78 years respectively. The values for diagnoses were 0 for no tinnitus diagnosis and 1 for tinnitus. Finally, for gender, 0 was assigned for male and 1 for female. Regression coefficients and associated p values were found for all the model variables and those variables with p values more than 0.05 for the constant were discarded. Thus, BC4000 (bone conduction at 4000 Hz), age, diagnosis and masker were not considered in the final model as the p values of their constants was more than 0.05, as shown in Tables 4 to 7.

**Table 4:** Logistic regression for BC4000

	Regression coefficient b	Standard error se(b)	Z	P
Constant	-0.09	0.08	-1.12	0.26
Bc4000_ind1	-0.15	0.11	-1.33	0.18
BC4000_ind2	-0.20	0.09	-2.12	0.03
BC4000_ind3	0.09	0.09	1.01	0.31

* Note: Bc4000_ind1, Bc4000_ind2 and Bc4000_ind3 represent bone conduction threshold quartiles of 25, 40 and 55 dB respectively.

**Table 5:** Logistic regression for age

	Regression coefficient b	Standard error se(b)	Z	P
Constant	-0.08	0.05	-1.49	0.13
Age_ind1	-0.13	0.08	-1.73	0.08
Age_ind2	-0.26	0.08	-3.48	0.00
Age_ind3	0.14	0.08	1.88	0.06

* Note: Age_ind1, Age_ind2 and Age_ind3 represent age quartiles of 60, 70 and 78 years respectively.

**Table 6:** Logistic regression for diagnosis

	Regression coefficient b	Standard error se(b)	Z	P
Constant	0.37	0.39	0.96	0.34
Diagnosis	-1.05	0.44	-2.37	0.02

**Table 7:** Logistic regression for masker

	Regression coefficient b	Standard error se(b)	Z	P
Constant	-0.41	0.25	-1.60	0.11
Masker_(No_masker, OTHERS)_	-0.91	0.50	-1.83	0.07

Due to data sparseness, it was not possible to incorporate all the keywords discovered by an analysis of the free text into a single model. However, a few significant keywords (meaningful words producing the highest chi-squared values, shown in Table 8), were included into the final model, along with all the categorical and numeric fields. These keywords were all acronyms where APPT stands for appointment, FTA for first time appointment, GP for general practitioner, MAP for processor amplification map (associated with cochlear implants), NFA for no follow-up-appointment and REV for hearing aid review.

**Table 8:** Logistic regression for keywords

	Regression coefficient b	Standard error se(b)	Z	P
Constant	-0.16	0.03	-5.63	0.00
APPT	0.06	0.15	0.37	0.71
FTA	-0.77	0.19	-4.05	0.00
GP	0.62	0.13	4.75	0.00
MAP	2.32	0.53	4.39	0.00
NFA	-0.93	0.32	-2.93	0.00
REV	0.12	0.10	1.12	0.26

## Results

### Principal component analysis (PCA)

The coefficients of the first PC (PC1) were all negative and approximately equal. This suggests that the main source of variation between the patients was simply the overall degree of hearing loss. The coefficients of the second PC (PC2) were negative for frequencies at or below 1000 Hz, but positive for higher frequencies, for both air and bone conduction, and thus differentiated patients according to whether they have a predominantly high frequency or low frequency hearing loss. The coefficients of the third PC (PC3) were positive for air conduction at all frequencies, but negative for bone conduction, showing a contrast between patients with and without an air-bone gap. The fourth component (PC4) was similar to the third, but corresponded to an air-bone gap at low frequencies. No clear patterns were seen for the fifth or subsequent principal components. The first four PCs corresponded to audiogram types frequently encountered in audiology clinics. The percentage of the overall variability in the data explained by the first four principal components respectively was 59.5, 13.4, 9.7, and 5.2, giving a total of 87.8%.

The thresholds corresponding to the first four PCs by using formula (1) are given in Table 9. The top row of each PC refers to the air conduction frequencies at 250, 500, 1000, 2000, 4000 and 8000 Hz respectively, while the second row refers to the corresponding bone conduction frequencies in the range 250 to 4000 Hz. The results are shown in Tables 10 to 12. The overall χ^2 ^(chi-squared value), calculated as the sum of the cells in Table 12, is 548.07, which for one degree of freedom gives p < 0.001, so audiogram type is clearly related to hearing aid type. Also, in Table 12, the (O - E)^2 ^/ E values which make the greatest contribution to the overall χ^2 ^value are those in the PCA1 and PCA3 columns. Thus, flat hearing loss (PCA1) audiograms without air-bone were associated with ITE aids and flat audiograms with additional air-bone gaps (PCA3) were associated with BTE aids. This result is in accordance with Stephens [3], who found that the fitting of ITE aids was limited in cases of severe hearing loss. Audiograms were also clustered using K-means clustering [4] which produced similar results to PCA, in that the mild to moderate hearing loss cluster was associated with ITE aids and the severe hearing loss cluster was associated with BTE aids. Thus, it was demonstrated that the audiogram is a factor influencing the choice of hearing aid type.

**Table 9:** The thresholds corresponding to the first four Principal Components

Principal Component (PC)	Frequency (in Hz)					
	250	500	1000	2000	4000	8000
PC1: Flat hearing loss	42	41	40	39	42	44
	45	45	42	42	45	
PC2: High tone sensorineural loss	37	38	48	69	82	79
	46	46	55	76	89	
PC3: Air-bone gap (flat)	78	77	75	71	75	76
	31	35	42	45	47	
PC4: Air-bone gap (predominant at low tone)	50	59	76	76	50	32
	29	55	82	85	52	

**Table 10:** Observed values (O)

Hearing aid type	PCA1	PCA2	PCA3	PCA4
ITE	2036	1341	476	75
BTE	1119	1166	1165	59

**Table 11:** Expected values (E)

Hearing aid type	PCA1	PCA2	PCA3	PCA4
ITE	1666.38	1324.12	866.73	70.77
BTE	1488.62	1182.88	774.27	63.23

**Table 12:** *(O-E)^2^/E *values

Hearing aid type	PCA1	PCA2	PCA3	PCA4
ITE	81.99	0.22	176.14	0.25
BTE	91.78	0.24	197.18	0.28

### Chi-squared test

The contingency table showing the relationship between gender and hearing aid type is shown in Table 12. The raw counts are given at the top of each cell, where for example there were 3196 male patients who wore BTE hearing aids. In each cell the Observed frequencies (*O*) are not enclosed in brackets, Expected frequencies (*E*) are in () and the quantity *(O - E)^2 ^/ E *is in []. The overall chi-squared value (the sum of the values in [] for all four cells) was 33.68, which for one degree of freedom is significant at *p < 0.001*. Males tended more to use ITE hearing aids and females tended more to use BTE hearing aids. For the relationship between hearing aid type and a diagnosis of tinnitus (ringing in the ear), the overall chi-squared value was 31.75, again significant at *p < 0.001 *for one degree of freedom. Patients with tinnitus tended more to wear ITE hearing aids. The relationship between the wearing of a tinnitus masker (a soothing sound source designed to drown out tinnitus) and hearing aid type, among patients diagnosed with tinnitus, had the overall chi-squared value of 17.16, which for one degree of freedom, was also significant at *p < 0.001*. The data for the cross-tabulation of hearing aid type and age produced the overall chi-squared value of 10.53, which for one degree of freedom, showed significance at *p < 0.001*. Mould type was also cross-tabulated with hearing aid type and the overall chi-squared value was 9844.18, which for 30 degrees of freedom was significant at *p < 0.001*. Thus all the category data types were significantly associated with hearing aid type. All the data in the patient records was used without considering confounding effects, where for example it might have been the choice of hearing aid type affecting the choice of mould, rather than vice versa. It is believed that this may have been the case, since many mould types never occurred in conjunction with one or the other hearing aid type.

The set of free-text keywords which tended to occur significantly more and less often (called positive and negative keywords respectively) in records where the patient wore either BTE or ITE aids are shown in Table 3. The association between these keywords and one or other type of hearing aid suggests the following: BTE aids were associated with high gain (amplification), e.g., be34, be36 and be52, and cases where changes had been made to the ear mould. ITE hearing aid types tended to use lacquer, had vents, required reshelling of ear impressions, had changes made to the hearing aid itself, were reviewed and the wearers were making progress.

### Logistic regression (LR) model

In Table 8, the part of the model which takes into account the occurrence or otherwise of the selected keywords in deciding which type of hearing aid to suggest is given. Using keywords alone, the relative likelihoods of the patient needing an ITE or a BTE aid are given by equation (3):

(3) $$\begin{array}{c} \text{log}\left[P\left(ITE/BTE\right)\right]=-0.16+0.06\left(APPT\right)-0.77\left(FTA\right)+0.62\left(GP\right)\\ +2.32\left(MAP\right)-0.93\left(NFA\right)+0.12\left(REV\right). \end{array}$$

As shown in Table 13, the logistic regression coefficient for gender was calculated. In Table 14, logistic regression values for air conduction (AC) at 250 dB are given for each quartile and in Table 15, predicted log odds are calculated using the regression coefficient values (b) from Table 14. Similarly, predicted log odds were calculated for AC500, AC1000, AC2000, AC4000, AC8000, BC250, BC500, BC1000, BC2000, gender and mould.

**Table 13:** Logistic regression for gender

	Regression coefficient b	Standard error se(b)	Z	P
Constant	-0.23	0.04	-5.93	0
Gender	0.16	0.05	3.08	0

**Table 14:** Logistic regression for AC250

	Regression coefficient b	Standard error se(b)	Z	P
Constant	-0.72	0.04	-17.23	0
AC250_ind1	0.54	0.07	8.15	0
AC250_ind2	1.29	0.07	17.26	0
AC250_ind3	2.18	0.12	17.91	0

* Note: Ac250_ind1, Ac250_ind2 and Ac250_ind3 represent Air conduction at 250 dB quartile of 40, 55 and 75 dB respectively.

**Table 15:** Predicted Log odds for AC250

AC250 group	Logistic regression equation	Predicted log odds
0<AC250< = 40	Log odds = b_constant_	-0.72
40<AC250< = 55	Log odds = b_constant _+ b_AC250_ind1_	-0.18
55<AC250< = 75	Log odds = b_constant _+ b_AC250_ind2_	0.57
75<AC250	Log odds = b_constant _+ b_AC250_ind3_	1.45

To show how the model works, a sample record from the test set is taken, as shown in Table 16. The attributes of this record are shown in the first column, and their values are shown in the second. Starting with a predicted log odds of 0 (meaning a BTE and ITE aid are assumed equally likely), the values of the attributes are examined in the record one by one, and add on the regression coefficient corresponding to that value of that attribute to the running total. The value of the first attribute, age is disregarded, but the next attribute gender has value "male". For "male", the regression coefficient is calculated to be -0.23, so the running total becomes 0 - 0.23 = -0.23. The relevant regression coefficients for each attribute are added in turn, ending with adding on -0.04 for the presence of the keyword "REV" in the free-text field. The final total of the regression coefficients is 10.1, which is the final log odds value, suggesting that it is much more likely that this patient would benefit most from a BTE hearing aid as opposed to an ITE aid.

**Table 16:** Logistic regression - worked example

Candidate variables (database record)	Actual values	Predicted log odds	Overall predicted log odds
Age	71	Not-significant	0
Gender	Male	-0.23	-0.23
AC250	75	0.57	0.34
AC500	70	0.72	1.06
AC1000	80	2.08	3.14
AC2000	90	1.19	4.33
AC4000	100	0.40	4.73
AC8000	100	0.09	4.82
BC250	40	-0.03	4.79
BC500	60	0.56	5.35
BC1000	65	0.56	5.91
BC2000	70	0.14	6.05
BC4000	70	Not-significant	6.05
Diagnosis	Tinnitus	Not-significant	6.05
Hearing aid type	BTE	To be found	6.05
Masker	No masker	Not-significant	6.05
Mould	2107	4.09	10.14
Free-text words	REV	-0.16+0.12 = -0.04	10.1

Testing of these logistic regression models showed that overall there was 81.64% agreement between the predictions of our model and the actual hearing aid chosen by the audiologist (as given in the "type" field) as shown in Table 17. The agreement rate was higher for patients fitted with ITE aids (86%) than for those fitted with BTE aids (76%). The results were analyzed according to precision, recall and F-measures [5] using equations (5), (7) and (8) respectively, as shown in Table 18. For comparison, a similar analysis using a Naïve Bayesian approach was performed, and 0.67, 0.76, and 0.71 were obtained as the precision, recall and F-score respectively for ITE and 0.66, 0.56 and 0.60 as the precision, recall and F-score respectively for BTE.

**Table 17:** Overall results

Results	Number of records	Percentage
Similar	1170	81.64
Not-similar	263	18.35
Total	1433	

**Table 18:** ITE/BTE aid Precision, Recall, F-score

	ITE	BTE
Precision	0.81	0.82
Recall	0.86	0.76
F-score	0.84	0.79

(4) $$P=\frac{Agreements\text{\_}of\text{\_}machine\text{\_}and\text{\_}human}{Total\text{\_}number\text{\_}in\text{\_}that\text{\_}category\text{\_}by\text{\_}machine}$$

(5) $$P_{ITE}=\frac{676}{\left(676+157\right)}=0.81$$

(6) $$R=\frac{Agreements\text{\_}of\text{\_}machine\text{\_}and\text{\_}human}{Total\text{\_}no.\text{\_}in\text{\_}that\text{\_}category\text{\_}in\text{\_}reality}$$

(7) $$R_{ITE}=\frac{676}{\left(676+106\right)}=0.86$$

(8) $$F_{ITE}=2\times \frac{P\times R}{P+R}=2\times \frac{0.81\times 0.86}{0.81+0.86}=0.84$$

In Table 19, 782 and 651 are the counts of ITE and BTE aids respectively in the human-annotated test data, while 833 and 600 are the counts of ITE and BTE aids respectively in the machine predicted results. The overall agreement is much better than random (50%), but the performance of a classifier should also be compared against the "simplest possible algorithm" [6]. In our case, this would be to assume that all the patients should be assigned the more commonly prescribed type of hearing aid. In our test set 782 out of 1433 patients in the test set were given ITE aids, so simply assigning all the patients this type of aid would provide 54.6% agreement, which is referred to as the ZeroR baseline.

**Table 19:** ITE/BTE aid predicted results

Machine results (logistic regression model)	Human (actual data)		
	**ITE**	**BTE**	**Total**
ITE	676 (86%)	106 (14%)	782
BTE	157 (24%)	494 (76%)	651
Total	833	600	1433

The theoretical upper bound of classifier performance is the inter-annotator agreement [2], in our case the rate at which two expert audiologists would assign the same hearing aid to the same patient. Unfortunately, we do not have data on this. Five-fold cross validation (repeated subsampling of the data to produce five non-overlapping test sets for an unbiased estimation of model accuracy) was performed. The overall similarity was in the range 82 to 85%, precision was in the ranges 0.79 to 0.87 for ITE and 0.82 to 0.85 for BTE, recall was 0.84 to 0.88 for ITE and 0.74 to 0.85 for BTE, and the F measure was 0.83 to 0.86 for ITE and 0.79 to 0.83 for BTE. For most of the cross-validation runs BC2000, BC4000, Diagnosis and Masker were discarded from the model, since these variables have p values of more than 0.05 for their constants. These results show that for each run, both the final model and the success rates were similar.

## Discussion

Although this LR model did not find age as a significant factor, Meredith and Stephens [7] have found that the ITE hearing aid presents handling problems only in subjects over 75 years of age. Dillon [8] also found that BTE aids are easier to operate as they are larger in size, and thus would be more popular with older people. The literature shows men and women preferring the two types for different reasons. Martin, et al. [9] found that more males choose ITE aids than females, because they perceive them to be a more advanced technology - though in reality the same makes and specifications are available in both styles, and neither model is more advanced than the other. They also found more females reporting that ITE aids are easier to handle than BTE. Mueller, et al. [10] found no difference in how embarrassed males and females feel about using a BTE aid. This LR model did not include diagnosis (as mentioned above for Table 6), although the authors previously found [11] that there was a significant association between the choice of BTE hearing aid and a diagnosis other than tinnitus (ringing in the ear), by using the chi-squared test. We also found, by using the chi-squared test that BTE hearing aids were atypical of tinnitus-with-masker. Other factors mentioned in the literature which could not be tested with this data are the greater cosmetic acceptability of the smaller ITE aids, comfort in wear, ease of use with spectacles, and sound quality [12].

## Conclusions

The associations between hearing aid type and audiogram type were confirmed by both the PCA/chi-squared and LR experiments described in this paper, and also by the authors' previous work on associations between words found in the database and hearing aid type, and the previous findings by audiologists [3]. These approaches will form the basis for an audiology decision support system, where unseen patient records would be presented to the system, and the relative probability that the patient should be fitted with an ITE aid as opposed to a BTE hearing aid would be returned. The advantage of these techniques for the combination of evidence is that it is easy to see which variables contributed to the final decision.

It is planned to validate these results by obtaining feedback from a professional audiologist, and by using an approach (Bayesian networks) which constructs model with interaction between variables. A major advantage of both Naïve Bayes and Logistic Regression is that they enable an explanation facility to be incorporated into any decision support tool, since it is easy to read back and see exactly which variables contributed exactly how much to the final decision of whether to fit a BTE aid or an ITE aid.

## Competing interests

The authors declare that they have no competing interests.

## Authors' contributions

MNA conducted the statistical analyses and drafted the manuscript together with MPO. MPO was project supervisor. Both authors contributed to the design and conception of this work. Both the authors have read and approved the final version of the manuscript.
